# It occurs after all: Attentional bias towards happy faces in the dot-probe task

**DOI:** 10.3758/s13414-020-02017-y

**Published:** 2020-03-31

**Authors:** Benedikt Emanuel Wirth, Dirk Wentura

**Affiliations:** Department of Psychology, Saarland University, Campus A2 4, D-66123 Saarbrücken, Germany

**Keywords:** Attentional bias, Dot-probe task, Spatial attention, Happy faces, Angry faces

## Abstract

Many studies have shown that not only threatening but also positive stimuli capture visual attention. However, in the dot-probe task, a common paradigm to assess attention to emotional stimuli, usually no bias towards happy faces occurs. Here, we investigated whether such a bias can occur and, if so, under which conditions. In Experiment 1, we investigated whether the bias is contingent on the simultaneous presentation of distractor stimuli with the targets. Participants performed a dot-probe task with either stand-alone targets or targets that were accompanied by distractors. We found an attentional bias towards happy faces that was not moderated by target type. To rule out perceptual low-level confounds as the cause of the bias towards happy faces, Experiments 2a and 2b comprised dot-probe tasks with inverted face cues. No attentional bias towards inverted happy faces occurred. In Experiment 3, we investigated whether a bias towards happy faces is contingent on a social-processing mode. Participants performed a dot-probe task with socially meaningful (schematic faces) or socially meaningless (scrambled schematic faces) targets. Again, a bias towards happy faces, which was not moderated by target type, occurred. In Experiment 4, we investigated the attentional bias towards happy faces when another highly relevant expression was present. Participants performed a dot-probe task with both happy and angry face cues. A significant attentional bias towards emotional faces occurred that did not differ between both cue emotions. These results suggest that happy faces are sufficiently relevant for observers to capture attention in the dot-probe task.

## Introduction

The human cognitive system is permanently confronted with an abundance of visual input. In order to cope with this overload of incoming information, selective attention determines which of the stimuli competing for access to the cognitive system are processed in a prioritized manner (Desimone & Duncan, 1995). A large body of psychological research has investigated the question as to whether specific stimuli have a natural advantage in this competition for attention or, in other words, whether specific stimuli capture visual attention. While traditional attention research has mainly focused on attentional capture by low-level perceptual stimulus features such as color, form, size, and orientation (e.g., Folk, Remington, & Johnston, 1992; Theeuwes, 1992; Treisman & Gelade, 1980; Wolfe, 1994), a smaller part of attention research has investigated the question as to whether specific stimuli can also capture attention due to higher-level features such as emotional valence (see Öhman & Mineka, 2001; Pool, Brosch, Delplanque, & Sander, 2016; Yiend, 2010, for reviews).

Traditionally, this line of research was focused on attentional bias towards negative (especially threatening) stimuli, for example snakes, spiders, and angry or fearful faces. This restriction to negative stimuli was most likely promoted by early theories of emotional attention. These theories claim that humans generally show an attentional bias towards threatening stimuli because it was an advantage during human phylogeny to preferentially attend to threatening stimuli and thus be able to react quickly in dangerous situations (Öhman, Flykt, & Esteves, 2001; Öhman & Mineka, 2001). More recent studies, however, have argued that human visual attention is not only biased towards threatening stimuli, but towards all stimuli that are relevant to the observer (e.g., Brosch, Pourtois, Sander, & Vuilleumier, 2011; Rothermund, Voss, & Wentura, 2008; Wentura, Müller, & Rothermund, 2014; Wentura, Rothermund, & Bak, 2000). This assumption is supported by a recent meta-analysis that shows that attention is also biased towards positive emotional stimuli. While positive emotional stimuli are certainly not threatening, they signal chances and opportunities and can therefore be equally relevant to observers.

Attentional biases have been found for a variety of positive emotional stimuli, for example baby faces (Brosch, Sander, Pourtois, & Scherer, 2008), erotica (Schupp et al., 2004), food-related stimuli (Tapper, Pothos, & Lawrence, 2010), and colors that have been associated with reward (Anderson, 2016; Anderson, Laurent, & Yantis, 2011; Müller, Rothermund, & Wentura, 2016; Wentura et al., 2014). Interestingly, evidence regarding the occurrence of an attentional bias towards happy faces has been inconclusive so far. On the one hand, some visual search studies have found evidence for an attentional bias towards happy faces (Becker, Anderson, Mortensen, Neufeld, & Neel, 2011; Savage, Lipp, Craig, Becker, & Horstmann, 2013). On the other hand, in the dot-probe task, one of the most common paradigms to measure biases towards emotional stimuli, attentional bias towards happy faces seems to be consistently absent (e.g., Baum, Schneider, Keogh, & Lautenbacher, 2013; Bradley et al., 1997; Cooper & Langton, 2006; Klumpp & Amir, 2009; Mogg & Bradley, 1999; Pourtois, Grandjean, Sander, & Vuilleumier, 2004). Most recently, Puls and Rothermund (2018) found no evidence for an attentional bias towards happy faces in six experiments comprising *N* = 275 participants in total.

Brosch et al. (2008), who are proponents of the relevance-captures-attention hypothesis, argue that happy faces might simply not be sufficiently relevant to capture visual attention because encountering a happy face does not require an urgent response – unlike encountering angry or fearful faces, which frequently requires a fight-or-flight response. Consistent with this hypothesis, Brosch et al. found an attentional bias in a dot-probe study towards baby faces. According to the authors, baby faces are a positive emotional stimulus class that can trigger a clear behavioral response (providing warmth and nurturance). In another dot-probe study, Cooper and Langton (2006) even found an attentional bias away from happy faces. The authors argue that participants might allocate attention towards the relatively more threatening stimulus class. If angry and neutral faces compete for attention, angry faces are the more threatening stimulus class. If neutral faces are presented simultaneously with happy faces, however, neutral faces are the more threatening stimulus class (see also Gronchi et al., 2018).

Nevertheless, the consistent absence of an attentional bias towards happy faces in the dot-probe task seems surprising since happy faces are a frequently encountered class of positive emotional stimuli that can convey lots of relevant signals for an observer, for example, safety, affiliation, and even sexual attraction. Accordingly, happy faces seem to affect several other cognitive processes. For example, numerous studies have found a clear recognition advantage for happy faces over other emotional expressions in emotion-categorization tasks (Calder, Young, Keane, & Dean, 2000; Calvo & Beltrán, 2013; Tottenham et al., 2009). Moreover, Rohr, Degner, and Wentura (2012) showed in a masked affective priming study that on a subconscious level, the distinction between happy faces and faces displaying other emotional expressions is particularly strong. Finally, in the approach-avoidance paradigm, happy faces can trigger motoric approach responses (e.g., Paulus & Wentura, 2014; Seidel, Habel, Kirschner, Gur, & Derntl, 2010).

The aim of the present study was to investigate whether an attentional bias towards happy faces can occur in the dot-probe task and, if so, under which conditions. As already mentioned, the dot-probe task is one of the most prominent paradigms to assess attentional biases towards emotional stimuli. It is a variant of the exogeneous spatial cueing paradigm (Jonides, 1981; Posner, Snyder, & Davidson, 1980). In the dot-probe task, participants have to classify a target stimulus that can appear in either of two screen positions (usually left or right of center) as fast as possible. The onset of the target is preceded by the presentation of two cue stimuli, one emotional (e.g., an angry face) and one neutral (e.g., a neutral face). Importantly, the position of the emotional cue stimulus is uncorrelated with the target position (i.e., the emotional cue is not predictive). Attentional bias towards the emotional cue stimulus is inferred if participants are faster to respond to the target when it appears in the location of the emotional stimulus (valid emotional cue) than when it appears in the opposite location (invalid emotional cue).^1^ The rationale of this approach is as follows. If the emotional cue captures attention, spatial attention will already be at the ideal location for target processing when the target appears in the same location, but not when it appears in the opposite location (for meta-analyses see Bar-Haim, Lamy, Pergamin, Bakermans-Kranenburg, & van IJzendoorn, 2007; Frewen, Dozois, Joanisse, & Neufeld, 2008).

Interestingly, while other paradigms reliably find attentional biases towards threatening stimuli in the general population (see Yiend, 2010, for a review), dot-probe studies usually find such a bias only in anxious, but not in non-anxious participants (for meta-analyses see Bar-Haim et al., 2007; Frewen et al., 2008). Consequently, it has been argued that attentional bias towards negative emotional stimuli in the dot-probe task might not be unconditional but contingent on top-down processes that are affected by participants’ trait anxiety (Puls & Rothermund, 2018) or by current task demands (Wirth & Wentura, 2018a, 2019). These considerations should be taken into account when investigating whether an attentional bias towards happy faces (i.e., positive emotional stimuli) can be reliably shown in the dot-probe task.

So, what could be the reasons that an attentional bias towards happy faces has been consistently absent in dot-probe studies – or, in other words, under which conditions could an attentional bias towards happy faces occur after all? First, we believe that the selection of an appropriate cue-target-onset asynchrony (CTOA) is critical. Most dot-probe studies employ a CTOA of 500 ms or even longer (Bar-Haim et al., 2007). However, stimulus-driven shifts in covert attention peak at 100–150 ms after stimulus onset (Müller & Rabbitt, 1989; Samuel & Kat, 2003). Accordingly, several authors recommend using SOAs of 200 ms or less in the dot-probe task to investigate shifts of covert attention, as longer SOAs possibly tap into shifts of overt attention (Cooper & Langton, 2006; Petrova, Wentura, & Bermeitinger, 2013; Stevens, Rist, & Gerlach, 2011; Weierich, Treat, & Hollingworth, 2008). Therefore, in the present study, we used a CTOA of 100 ms throughout all experiments.

Second, we believe that it is critical to employ unconfounded cue stimuli. Emotional and neutral faces do not only differ regarding their emotional valence, but also regarding their perceptual low-level properties. Especially happy faces are more salient than neutral faces because of the high luminance that is caused by exposed teeth in happy expressions (Calvo & Nummenmaa, 2008; Horstmann, Lipp, & Becker, 2012). In a dot-probe study, we recently showed that the assessment of attentional bias towards angry faces is severely distorted if angry face cues with exposed teeth are employed (Wirth & Wentura, 2018b). Therefore, throughout our experiments, we employed happy face cues with concealed teeth (i.e., with closed mouths).

**Fig. 1:**
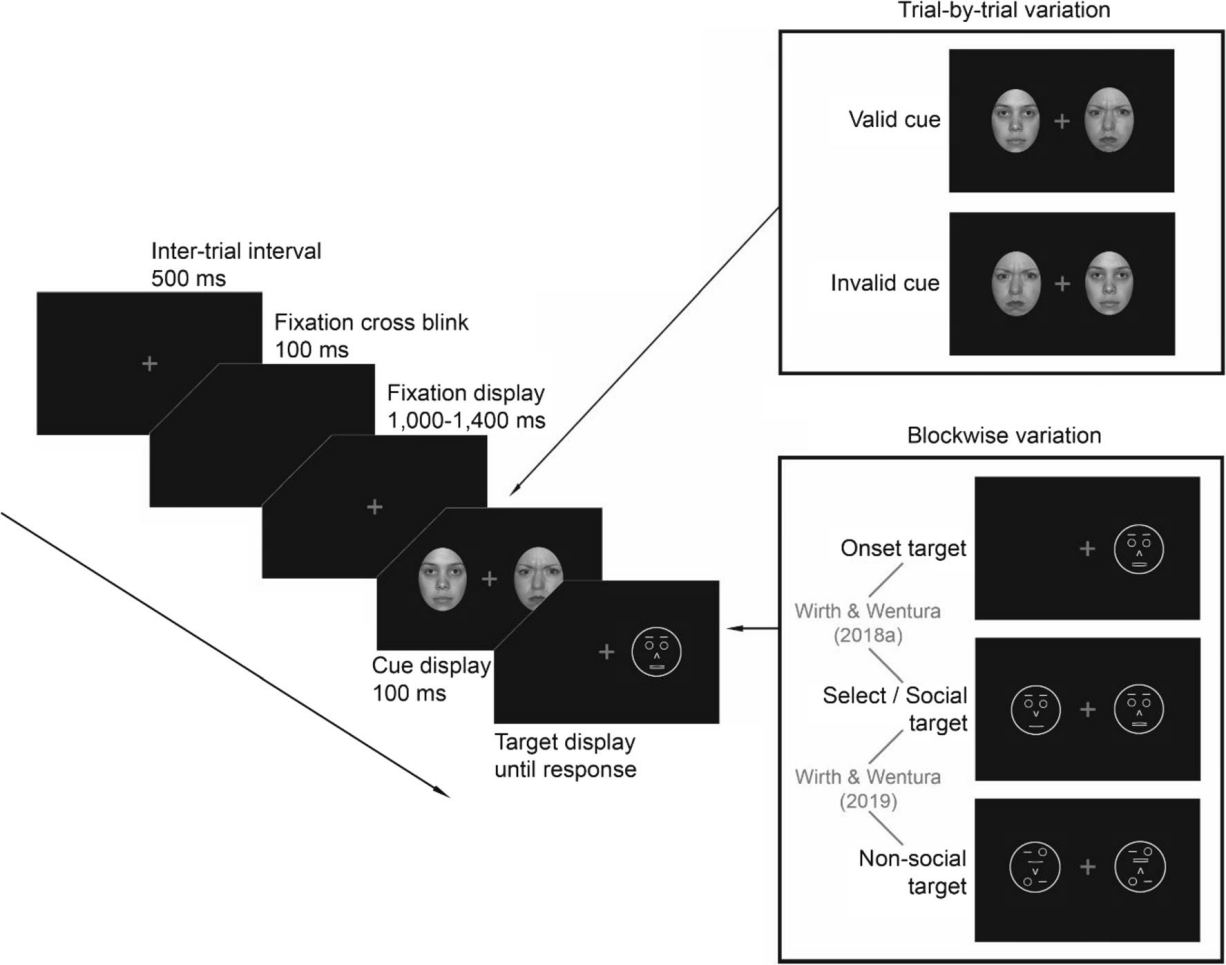
Schematic illustration of a typical trial and the design of our previous studies. Participants had to find the stimulus with the horizontal double line (here: the right one) and report the direction of its nose (arrow). A valid trial is depicted here as the target is in the same position as the angry face. For the sake of visibility, proportions are not true to scale

Third, attentional bias towards happy faces in the dot-probe task might be contingent on specific top-down processes. We recently showed in two dot-probe studies that attentional bias towards angry faces is contingent on top-down processes in non-anxious participants (see Fig. 1 for a schematic illustration of the study designs). In one experiment (Experiment 1 in Wirth & Wentura, 2019), participants performed a dot-probe task with photographic neutral and angry face cues and – importantly – two different target conditions that were markedly different from typical dot-probe studies. In the *social target condition,* two schematic faces were presented left and right of fixation. One schematic face had an open mouth (indicated by a horizontal double line) and one schematic face had a closed mouth (indicated by a single horizontal line). Participants were asked to find the schematic face with the open mouth (target) and to indicate whether its nose was pointing up or down while ignoring the schematic face with the closed mouth (distractor). *In the non-social target condition,* scrambled schematic faces (with the mouth above the nose, one eye below the nose, etc.) were presented on the target display. These scrambled schematic faces conveyed the impression of complex, meaningless patterns and, thus, participants’ task was to find the pattern with the horizontal double line and indicate whether the arrow in this pattern was pointing up or down. A significant attentional bias towards angry face cues occurred only in the social target condition, but not in the non-social target condition. Therefore, the bias seemed to occur only when participants had to perform a task that required processing of socially meaningful stimuli, that is, when a top-down social-processing mode was activated.

However, another experiment (Experiment 2 in Wirth & Wentura, 2018a) showed that simply using socially relevant target stimuli is not sufficient to trigger an attentional bias towards angry face cues. In this experiment, participants again performed a dot-probe task with two target conditions. The *select target condition* was identical to the *social target condition* of the other experiment. Thus, participants were presented with two schematic faces and had to categorize the one with an open mouth. In the *onset target condition,* however, only a single schematic face was presented (i.e., the target) that participants had to classify. A significant attentional bias towards angry face cues occurred only in the select target condition, but not in the onset target condition. Thus, it seems that attentional bias towards angry face cues occurs only when targets have to compete for attention with distractor stimuli, but not when participants are searching for onset-singleton targets (see also Gaspelin, Ruthruff, & Lien, 2016). Thus, in the present experiments, we investigated whether attentional bias towards happy faces in the dot-probe task is contingent on similar top-down processes, that is (1) a non-singleton search mode and (2) the activation of a social-processing mode. As in our experiments with angry faces, we again used schematic faces as target stimuli for this purpose.

## Experiment 1

In Experiment 1, we investigated whether attentional bias towards happy face cues in the dot-probe task occurs only if the target on a given trial has to compete for attention with a simultaneously presented distractor stimulus (i.e., if it is not an onset-singleton). Participants performed a dot-probe task with happy face cues and two target conditions. Both target conditions used socially meaningful stimuli (schematic faces). However, in the onset target condition, only a single schematic target face was presented that had to be classified. In the select target condition, two schematic faces were presented, and participants had to select the target face based on a socially relevant dimension. Note that Wirth and Wentura (2018a) found an attentional bias towards angry faces only in the select target condition.

### Method

#### Participants

Seventy-eight university students were paid €6 for their participation. Four participants were excluded from all further analyses, three of them because their overall accuracy in at least one of the experimental blocks was more than 3 interquartile ranges below the first quartile of the overall distribution and one because the average response time (RT) in one of the experimental blocks was more than 3 interquartile ranges above the third quartile of the overall distribution (Tukey, 1977). Of the remaining *N* = 74 participants, 56 were female; age ranged from 18 to 34 years (*M* = 24.7 years, *SD* = 3.5). All participants reported normal or corrected-to-normal vision and provided informed consent prior to testing.

The sample size was determined according to the following considerations: We aimed to have sufficient power to detect (1) an attentional bias towards happy faces and (2) a potential interaction of cue validity and target type (i.e., a potential magnitude difference between attentional bias in the onset vs. select target conditions). We based the size estimates for both effects on our previous study (Wirth & Wentura, 2018a). In that study, we found attentional biases towards angry faces with effect sizes of *d*_*Z*_ = 0.49 and *d*_*Z*_ = 0.34 (for Experiment 1 and Experiment 2, respectively) under conditions that were identical to the select target condition of the present experiment. Regarding the interaction effect, Experiment 2 of our previous study showed a moderation of the attentional bias towards angry faces by target type with an effect size of *d*_*Z*_ = 0.33 According to Cohen (1988), such an effect can be considered in-between “small” (*d*_*Z*_ = 0.2) and “medium” (*d*_*Z*_ = 0.5). We chose to base our power considerations on assumptions that are slightly more conservative. A power analysis using G*Power (Faul, Erdfelder, Lang, & Buchner, 2007) showed that a sample size of *N* = 74 allowed us to detect effects of *d*_*Z*_ = 0.29 with a probability of 1 - β = .80, given an α-value of α = .05 (one-tailed).

#### Design

We employed a 2 (*cue validity:* valid cue vs. invalid cue) × 2 (*target type:* onset target vs. select target) design with *cue validity* as a trial-by-trial within-subjects factor and *target type* as a blockwise within-subjects factor.

#### Materials

As cues, we used photographs of eight female and eight male individuals showing happy and neutral expressions that were taken from the NimStim set of facial expressions (Tottenham et al., 2009). Since exposed teeth are a strong perceptual confound of happy expressions that can potentially distort dot-probe effects (Wirth & Wentura, 2018b), we only employed happy faces with closed mouths in the present study. Thus, the intensity of the emotional expression is rather moderate in these faces. Using Adobe Photoshop (Adobe Systems Inc., San Jose, CA, USA), all stimuli were cropped into a standard oval shape concealing hair and external features and were converted to grayscale (see Fig. 1).

#### Procedure

The study was conducted on five PCs equipped with 17-in. CRT monitors using a resolution of 1,024 × 768 Pixels, a refresh rate of 100 Hz, and a color depth of 32 bit. The experimental routine was programmed using Psychtoolbox-3 (Kleiner, Brainard, & Pelli, 2007) for Matlab 2014a (Mathworks, Natick, MA, USA).

**Fig. 2:**
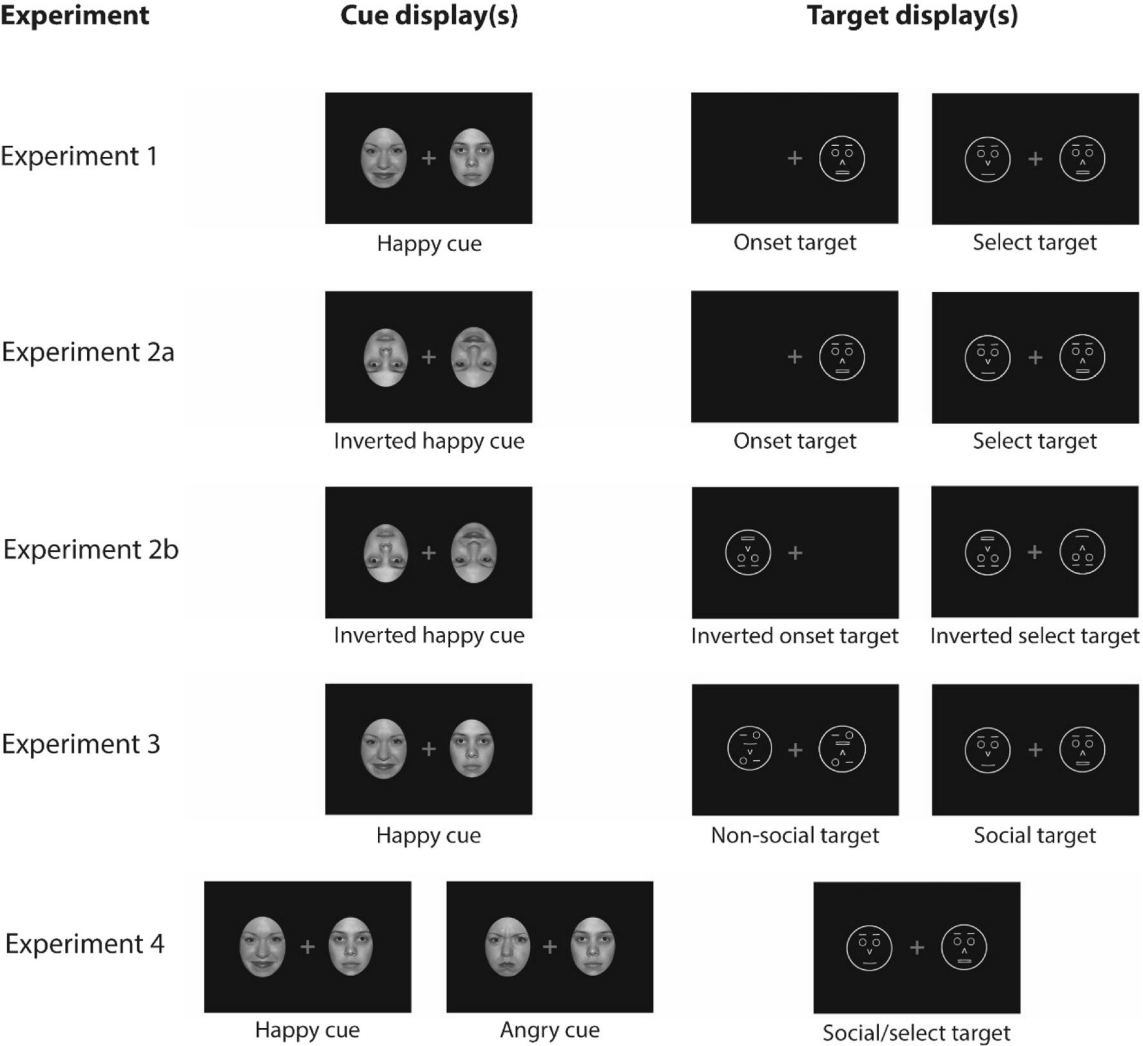
Schematic illustration of the cue and target manipulations of all experiments. In Experiments 1*–*3 target displays were experimentally varied whereas in Experiment 4 the emotion of the photographic face was experimentally varied. For Experiment 2a, a valid trial is depicted as the (inverted) happy face cue is in the same location as the target. For the remaining experiments, invalid trials are depicted as the emotional face cues are in the opposite location of the targets. For the sake of visibility, proportions are not true to scale

At the beginning of the session, participants were seated in an individual testing booth, approximately 65 cm from the monitor. After completion of the consent form, they were presented with an instruction screen explaining the experimental procedure. Temporal parameters were identical to our previous dot-probe studies with angry faces (see Fig. 1). Figure 2 illustrates the experimental variation of the target stimuli. Throughout the procedure, a gray fixation cross was presented on a black background to maintain participants’ focus at the center of the screen. To indicate the beginning of a trial, the fixation cross blinked for 100 ms. The fixation cross then remained on-screen for a variable interval (chosen randomly from the set 1,000, 1,100, 1,200, 1,300, or 1,400 ms) to avoid any anticipatory effects. Subsequently, two photographic face cues, one happy and one neutral, were presented to the left and right of the fixation cross for 100 ms. Each face had a size of 4.5 × 6.2 cm (4.0 × 5.5°); the center-to-center distance between the faces was 11.1 cm (9.8°). Immediately after the offset of the cues, the target display was presented until a response was given.

In the onset target condition, the target was a single white line drawing of a schematic face with a neutral expression. The schematic target face was presented either in the left or in the right screen position. Participants’ task was to indicate as fast as possible whether the nose of the schematic face (which was symbolized by an arrowhead) was pointing up or down. Thus, the target in this condition was an onset singleton. In the select target condition, two schematic faces (also with neutral expressions) were presented during the target display, one target face with an open mouth (symbolized by a horizontal double line) and a schematic distractor face with a closed mouth (symbolized by a single horizontal line). Participants had to indicate the direction of the nose of the target face while ignoring the distractor face. Thus, the target had to be selected based on a socially relevant dimension (i.e., an open mouth) in this condition before it could be classified.

The schematic faces had a size of 2.8 × 2.8 cm (2.5 × 2.5°) and the center-to-center distance between them was 11.1 cm (9.8°). In the select target condition, nose/arrow directions of target and distractor stimuli were uncorrelated, that is, the nose/arrow of the target stimulus pointed in the same direction as the nose/arrow of the distractor stimulus on 50% of the trials and in the opposite direction on the remaining trials (this was varied orthogonally to the other experimental factors). Participants were asked to respond as fast as possible by pressing the “t” key for “up,” or the “v” key for “down,” on a standard German QWERTZ keyboard. Importantly, on half the trials, the target stimulus appeared at the location of the happy face cue (valid cue) and on the remaining trials it appeared at the location of the neutral face cue (invalid cue). Each response was followed by a 500-ms inter-trial interval. If participants made an error or took longer than 1,500 ms to submit a response, they received a 1,000-Hz warning tone of 500-ms duration via headphones.

The experiment comprised 448 trials and lasted approximately 35 min. Trials were presented in two blocks consisting of 224 trials each – one with onset targets and one with select targets, in a counterbalanced order. Within each block, a self-paced break was included after 112 trials. At the beginning of each block, participants were presented with 32 training trials that were not included in data analysis. These training trials used face cues of individuals that were not presented during the main trials.

At the end of the experiment, participants completed the trait-anxiety scale of the State-Trait Anxiety Inventory (STAI; Laux, Glanzmann, Schaffner, & Spielberger, 1981). Since it is the standard in dot-probe research with threatening stimuli (see Introduction), we assessed participants’ trait anxiety in our earlier experiments with angry (i.e., threatening) faces (Wirth & Wentura, 2018a, 2018b). Therefore, we decided to assess participants’ trait anxiety in the present experiment as well, although we did not expect any correlations between attentional bias towards happy faces and anxiety. Note that if we had found correlations of trait anxiety with attentional bias towards angry faces in our previous studies, it would have been mandatory to assess the correlation between anxiety and attentional bias towards happy faces as well to check for discriminant validity.

### Results

Average classification accuracy was *M* = 97.6% (*SD* = 1.8). For the RT analysis, RTs below 150 ms were excluded, as were RTs more than 1.5 interquartile ranges above the third quartile of the individual participant’s distribution (separately for both experimental blocks; Tukey, 1977). This led to the exclusion of 2.3% of all trials with correct responses. After outlier removal, average individual RTs for correct responses ranged from *M* = 544 to *M* = 880 ms (grand mean was *M* = 670 ms, *SD* = 67). Table 1 shows average RTs as a function of the experimental factors.

**Table 1:** Mean response times (RTs) and cueing scores (in ms) of Experiment 1 as a function of target type and cue validity

Target type	Cue validity		
	Valid	Invalid	Cueing score
Onset target	606 (97.9)	610 (98.2)	4 [1, 7]
Select target	729 (97.1)	734 (97.0)	5 [0, 9]
Overall	668 (97.5)	672 (97.6)	4 [2, 7]

*Note.* Accuracy rates (in %) are given in parentheses, 95% confidence intervals are given in brackets, cueing score = RT_invalid_ – RT_valid_, deviations between the differences of mean RTs and the cueing scores are due to rounding

We conducted a 2 × 2 within-subjects ANOVA with the factors *target type* (onset target vs. select target) and *cue validity* (valid cue vs. invalid cue) and (correct) RTs as the dependent variable. The analysis revealed significant main effects of *target type*, *F*(1, 73) = 451.12, *p* < .001, η_p_^2^ = .861, which reflects faster RTs in the onset target condition (*M* = 608 ms, *SD* = 60) than in the select target condition (*M* = 731 ms, *SD* = 82). This result is not surprising because in the select target condition, participants had to select the target stimulus first before they could categorize it. More importantly, there was also a significant main effect of *cue validity, F*(1, 73) = 10.15, *p* = .002, η_p_^2^ = .122, which reflects faster RTs for valid trials (*M* = 668 ms, *SD* = 68) than for invalid trials (*M* = 672 ms, *SD* = 67).

**Fig. 3:**
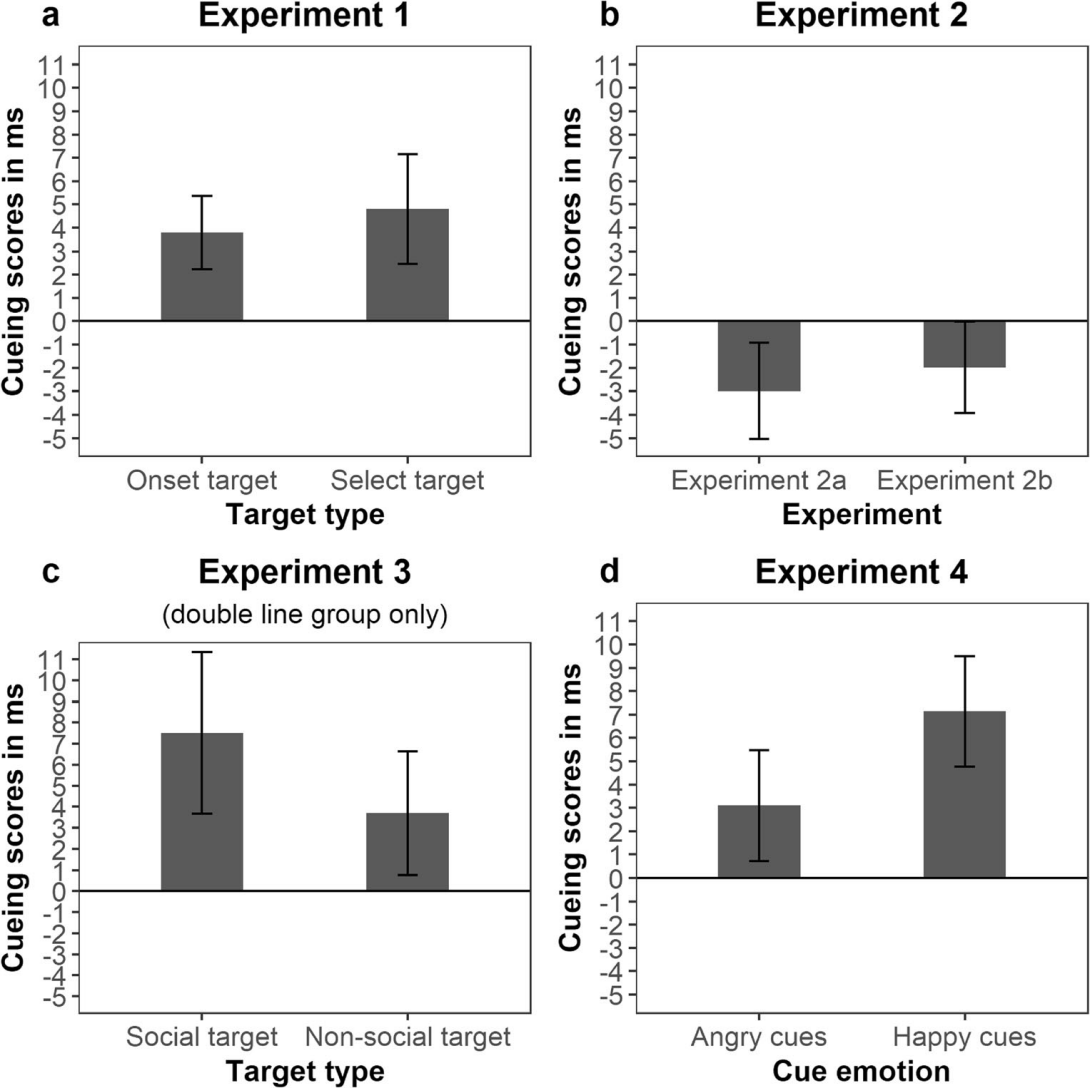
Mean cueing scores (RT_invalid_ – RT_valid_) in all experiments. Error bars depict ±1 standard error of the mean (SEM). For an illustration of the distribution of individual cueing scores, readers are referred to Fig. 4 in the Appendix

The *target type* × *cue validity* interaction did not reach significance, *F*(1, 73) < 1. Nevertheless, for the sake of completeness, we calculated separate cueing scores for both target conditions by subtracting average individual RTs of valid trials from average individual RTs of invalid trials. Thus, a positive cueing score in a given condition reflects an attentional bias towards happy faces in that condition. As can be seen in Fig. 3a, cueing scores in the onset target condition (*M* = 4 ms, *SE* = 2) and in the select target condition (*M* = 5 ms, *SE* = 2) were of almost equal size (which corresponds to the non-significant *target type* × *cue validity* interaction in the ANOVA). Holm-Bonferroni corrected *t*-tests showed that cueing scores both in the onset target condition, *t*(73) = 2.43, *p* = .018, *d*_*Z*_ = 0.28, and in the select target condition, *t*(73) = 2.05, *p* = .044, *d*_*Z*_ = 0.24, differed significantly from zero. The size of the combined cueing effect across both conditions was *d*_*Z*_ = 0.37. As expected, participants’ trait anxiety as assessed with the STAI did not significantly correlate with cueing scores in the onset target condition, *r*(71) = -.178, *p* = .131, in the select target condition, *r*(71) = .178, *p* = .133, or with the combined cueing score across both conditions, *r*(71) = .054, *p* = .652.^2^

Attentive readers might ask whether in the select target condition, a target-distractor congruency effect, in terms of a response facilitation if both noses point in the same direction (congruent targets) and a response inhibition if both noses point in different directions (incongruent targets) occurred. This was indeed the case: Responses were significantly slower in trials with incongruent nose directions than in trials with congruent nose directions, *t*(73) = 6.08, *p* < . 001, *d*_*z*_ = 0.71. Keeping the nose direction of the targets and of the distractors uncorrelated was a technical necessity: If target-nose direction and distractor-nose direction were correlated (e.g., target nose and distractor nose always point in opposite directions), one could simply solve the task by focusing on one screen location and deciding whether the stimulus in that location is the target or the distractor). Since target congruency was orthogonalized in regard to the other experimental factors, the effects of cue validity and target type are not confounded by target-distractor congruency.

### Discussion

In Experiment 1, we found a significant attentional bias towards happy faces in the dot-probe task. Interestingly, this bias was equally large for the onset target condition and the select target condition. Therefore, in contrast to the effects we previously found for angry faces (Wirth & Wentura, 2018a), it seems that the attentional bias was not moderated by the presence of distractor stimuli on the target display.

It should be noted that the absence of a moderation of the cueing scores by target type yields a problem for the interpretation of the results. Especially in this case, one could argue that the bias was not caused by the emotional character of the happy face cues, but by perceptual low-level confounds of the happy face cues. More specifically, it is possible that the happy face cues we employed in our experiment were more salient than their neutral counterparts and therefore captured visual attention. We conducted Experiments 2a and 2b as controls to rule out this potential explanation for the results of Experiment 1.

## Experiment 2a

Since we only included happy faces with non-exposed teeth in Experiment 1, we assumed that the bias was genuinely caused by the emotional character of the happy face cues and not by confounded salient low-level characteristics of these faces. Nevertheless, in order to rule out the latter possibility, inverted face cues (happy and neutral) were presented in Experiment 2a. Inversion disrupts the holistic processing of faces (see Valentine, 1988, for a review on the effect) and therefore severely impairs the recognition of emotional expressions. In contrast, perceptual low-level characteristics of faces are still preserved when faces are inverted. Thus, if the attentional bias towards upright happy faces in Experiment 1 was caused by low-level confounds, an attentional bias should also occur towards inverted happy faces. If, however, the attentional bias occurred due to the emotional character of the happy faces, no attentional bias towards inverted happy faces should occur.

Since we did not expect an attentional bias towards inverted happy faces and standard null hypothesis statistical testing (NHST) does not offer the possibility to quantify the evidence in favor of null effects, we chose to apply an alternative statistical procedure that allows provision of evidence for null effects, namely *Sequential Bayes Factors* (for a detailed description of the procedure, see Schönbrodt, Wagenmakers, Zehetleitner, & Perugini, 2017). The Bayes factor is a Bayesian model selection measure that quantifies the probability of given data under *H*_0_ relative to the probability of the data under *H*_1_. For example, if one assumes that *H*_0_ and *H*_1_ are equally probable a priori, a Bayes factor of *BF*_01_ = 10 after data collection indicates that the data are ten times as likely to have occurred under *H*_0_ than under *H*_1_. Conversely, a Bayes factor of *BF*_01_ = 1/10 indicates that the data are ten times as likely to have occurred under the *H*_1_ than under the *H*_0_ (see Wagenmakers, Wetzels, Borsboom, & van der Maas, 2011, for interpretation guidelines for specific Bayes factor values). In the sequential Bayes factors procedure, data can be monitored while they are collected. That is, after an initial number of participants have been tested, their data are inspected, and the corresponding Bayes factor is calculated. If the Bayes factor reaches a pre-defined value in favor of *H*_0_ or *H*_1_, sampling is stopped, and the favored hypothesis is accepted. If the Bayes factor does not reach either the *H*_0_ boundary or the *H*_1_ boundary, sample size is increased in pre-defined steps until the Bayes factor reaches one of the boundaries. Since this procedure can lead to undesirably large sample sizes (because the Bayes factor meanders between the *H*_0_ boundary and the *H*_1_ boundary), a maximum number of sampled participants is also specified.

Following recommendations by Schönbrodt et al. (2017), we defined the following parameters a priori.^3^ We set the *H*_0_ boundary at *BF*_01_ = 6 and the *H*_1_ boundary at *BF*_01_ = 1/6 (i.e., *BF*_10_ = 6), respectively, and used a scale parameter of *r* = 1 for the JZS *H*_1_ effect size prior. Moreover, we set the initial sample size at *n* = 40 participants. If the Bayes factor does not reach one of the boundaries after 40 participants, we will continue data collection and observe the Bayes factor after each day of testing. If the Bayes factor does not reach a boundary after 80 participants have been tested, we will terminate data collection.

### Method

#### Participants

Forty-nine university students were paid €6 for their participation. Unfortunately, due to the a priori exclusion criteria that were based on the distribution of Experiment 1, seven participants had to be excluded from all further analyses.^4^ Of the remaining *N* = 42 participants, 33 were female; age ranged from 18 to 33 years (*M* = 23.2, *SD* = 3.3). All participants reported normal or corrected-to-normal vision and provided informed consent prior to testing.

#### Design

We calculated a cueing score by subtracting average individual RTs on valid trials from average individual RTs on invalid trials. This cueing score was then compared to zero using Bayesian one-sample t-tests.

#### Materials and procedure

The same materials as in Experiment 1 were used. The experimental procedure was also identical to Experiment 1, apart from one exception. On the cue displays, happy and neutral faces were not presented upright, but inverted (see Fig. 2 for a schematic illustration).

### Results

Average classification accuracy was *M* = 97.3% (*SD* = 2.1). For the exclusion of RT outliers, the same criteria as in Experiment 1 were applied. This led to the exclusion of 2.1% of all trials with correct responses. After outlier exclusion, average individual RTs for correct responses ranged from *M* = 583 to *M* = 862 ms (grand mean was *M* = 686 ms, *SD* = 72).

We first checked the Bayes factor after data of 40 participants had been collected. Using a one-sided Bayesian t-test (i.e., *H*_1_ assumes that the attentional bias towards inverted happy faces is larger than zero), we obtained a Bayes factor of *BF*_01_ = 26.39 (i.e., strong evidence in favor of *H*_0_). According to our pre-defined criteria, we could have stopped data sampling at that point. However, the cueing score produced by inverted happy faces was actually negative (*M* = -5 ms, *SD* = 12). Therefore, a two-sided Bayesian t-test showed anecdotal support for *H*_1_ with a Bayes factor of *BF*_01_ = 0.49 (i.e., *BF*_10_ = 2.04). Moreover, this negative cueing score was significant according to traditional NHST, *t*(39) = 2.48, *p* = .017, *d*_*Z*_ = 0.39. Therefore, we decided to continue data sampling to rule out the possibility that this negative cueing effect was actually meaningful.

After one more day of testing, data from two additional participants had been collected (i.e., the sample consisted of *N* = 42 participants). Using a one-sided Bayesian t-test, we again obtained strong evidence in favor of *H*_0,_*BF*_01_ = 19.12. While the cueing score was still negative (see Fig. 3b), a two-sided Bayesian t-test no longer showed anecdotal support for *H*_1_, *BF*_01_ = 3.06. Moreover, the negative cueing score produced by inverted happy faces was not significant anymore according to NHST, *t*(41) = 1.45, *p* = .156, *d*_*Z*_ = 0.22. Therefore, we decided to terminate data collection at this point. Table 2 shows average RTs as a function of the experimental factors. As can be seen from the table, there was no interaction between target type and cue validity.

**Table 2:** Mean response times (RTs) and cueing scores (in ms) of Experiment 2a as a function of target type and cue validity

Target type	Cue validity		
	Valid	Invalid	Cueing score
Onset target	624 (98.0)	623 (98.0)	-2 [-2, 2]
Select target	751 (97.0)	747 (96.3)	-4 [-11, 3]
Overall	688 (97.5)	685 (97.1)	-3 [-7, 1]

*Note.* Accuracy rates (in %) are given in parentheses, 95% confidence intervals are given in brackets, cueing score = RT_invalid_ – RT_valid_, deviations between the differences of mean RTs and the cueing scores are due to rounding

### Discussion

We conducted Experiment 2a as a control to rule out the possibility that the attentional bias towards happy faces that we found in Experiment 1 was caused by perceptual low-level confounds of the happy faces presented in Experiment 1. Therefore, the same face cues were presented inverted in Experiment 2a. We did not find evidence for an attentional bias towards inverted happy faces in Experiment 2a. Since face inversion disrupts holistic processing of faces but preserves perceptual low-level properties, this result suggests that the attentional bias towards happy faces in Experiment 1 was genuinely caused by the emotional character of happy faces and not by their perceptual properties. However, inverting the face cues does not only disrupt holistic processing but also the spatial mapping of specific cue and target features. The mouth region was the target-defining feature of the schematic faces in Experiments 1 and 2a (at least in the select target condition). Thus, it is possible that participants showed an attentional bias towards happy face cues in Experiment 1 because happy faces have perceptually larger mouth areas than neutral faces and the mouth area of the face cues spatially overlapped with the target-defining mouth area of the schematic faces. Due to the inversion of the cues in Experiment 2a, however, the mouth regions of the face cues and the schematic faces were no longer presented in the same spatial position, which could explain the absence of an attentional bias towards inverted happy faces in Experiment 2a. To rule out this possibility, we conducted Experiment 2b.

## Experiment 2b

Experiment 2b was identical to Experiment 2a with the only exception that not only the photographic face cues were inverted, but also the schematic target (and distractor) faces. If the attentional bias towards happy faces in Experiment 1 was merely caused by spatial overlaps between the mouth regions of the face cues and the target stimuli, an attentional bias towards inverted happy faces should occur in Experiment 2b. We again used the sequential-Bayes-factors procedure with the same parameters as in Experiment 2a to establish whether an attentional bias towards inverted happy faces was present or not.^5^

### Method

#### Participants

Forty-six university students received €6 or course credit as compensation for their participation. Note that we were again faced with the problem of pre-defining a viable criterion for the exclusion of participants since there is no “final sample” in testing procedures involving sequential Bayes factors. Unlike in Experiment 2a, we could not simply use the absolute cut-off values from Experiment 1 because the inversion of the target stimuli fundamentally changed the target-categorization task. Therefore, we decided to derive cut-off values from the initial sample of 40 participants: Participants were excluded if their accuracy in either of the two (or both) target conditions was more than three interquartile ranges below the first quartile of the distribution of the 40 participants initially tested. Furthermore, participants were excluded if their mean RT in either of the two (or both) target conditions was more than three interquartile ranges above the third quartile of the distribution of the 40 participants initially tested. Due to these criteria, six participants had to be excluded from all further analyses. Of the remaining *N* = 40 participants, 30 were female; age ranged from 19 to 34 years (*M* = 24.5, *SD* = 4.1). All participants reported normal or corrected-to-normal vision and provided informed consent prior to testing.

#### Design

Again, we calculated a cueing score by subtracting average individual RTs on valid trials from average individual RTs on invalid trials, which was then compared to zero using Bayesian one-sample t-tests.

#### Materials and procedure

The material and experimental procedure were identical to Experiment 2b, apart from one exception. Not only the photographic face cues were inverted, but also the schematic target (and distractor) faces (see Fig. 2 for a schematic illustration).

### Results

Average classification accuracy was *M* = 96.6% (*SD* = 2.1). For the exclusion of RT outliers, the same criteria as in Experiments 1 and 2a were applied. This led to the exclusion of 2.9% of all trials with correct responses. After outlier exclusion, average individual RTs for correct responses ranged from *M* = 571 to *M* = 821 ms (grand mean was *M* = 656 ms, *SD* = 65).

The average cuing score is depicted in Fig. 3b. We first checked the Bayes factor after data of 40 participants had been collected. Using a one-sided Bayesian t-test (i.e., *H*_1_ assumes that the attentional bias towards inverted happy faces is larger than zero), we obtained a Bayes factor of *BF*_01_ = 15.31 (i.e., strong evidence in favor of *H*_0_).^6^ According to our pre-defined criteria, we stopped data sampling at that point and accepted the null hypothesis. Table 3 shows average RTs as a function of the experimental factors. There was no evidence for an interaction between target type and cue validity, *BF*_01_ = 3.50.

**Table 3:** Mean response times (RTs) and cueing scores (in ms) of Experiment 2b as a function of target type and cue validity

Target type	Cue validity		
	Valid	Invalid	Cueing score
Onset target	582 (97.4)	583 (97.4)	1 [-4, 6]
Select target	733 (96.1)	728 (95.6)	-5 [-11, 2]
Overall	658 (96.7)	656 (96.5)	-2 [-6, 2]

*Note.* Accuracy rates (in %) are given in parentheses, 95% confidence intervals are given in brackets, cueing score = RT_invalid_ – RT_valid_, deviations between the differences of mean RTs and the cueing scores are due to rounding

### Discussion

In Experiment 2b, we aimed to rule out the possibility that the attentional bias towards happy face cues in Experiment 1 occurred due to a spatial overlap between the mouth regions of the happy face cues and the target-defining mouth regions of the schematic faces presented during the target display. To this end, we presented both inverted photographic faces on the cue display and inverted schematic faces on the target display because this set-up preserves the spatial overlap between specific cue and target features (especially of the mouth region). However, no reliable attentional bias towards inverted happy face cues occurred in Experiment 2b. Thus, the results of both Experiment 2a and Experiment 2b suggest that the bias towards upright happy face cues found in Experiment 1 was caused by emotional valence and not by perceptual low-level confounds.

## Experiment 3

The results of Experiments 1, 2a, and 2b suggest that an attentional bias towards happy faces can occur in the dot-probe task under specific conditions. However, this bias was not contingent on the presentation of distractor stimuli during the target display – unlike attentional bias towards angry faces as we have previously shown (Wirth & Wentura, 2018a). So why did a bias towards happy faces occur in Experiment 1 while other dot-probe studies did not find such a bias? One explanation might be that attentional bias towards happy faces is not contingent on a competition between target and distractor stimuli for attention but on the activation of a social-processing mode. Previously, we have argued that a competition between target and distractor stimuli is a necessary precondition for the activation of a social-processing mode since stand-alone targets do not need to be processed in terms of their social character (Wirth & Wentura, 2019); that is, when a stand-alone target is presented, participants can merely focus on the arrow that symbolizes the nose of the schematic face and ignore the remaining features of the schematic face. Thus, no social-processing mode might be activated in participants if face-like stimuli are presented as stand-alone targets. However, face perception seems to be a mandatory process (at least to some degree; see Palermo & Rhodes, 2007, for a review). That is, perceived faces cannot be “unseen” and are processed automatically. In order to investigate whether attentional bias towards happy faces is contingent on a social-processing mode but not on target competition, we conducted Experiment 3 similar to our previous study with angry faces (Wirth & Wentura, 2019). That is, participants performed a dot-probe task either with socially meaningful targets (schematic faces) or with meaningless targets (scrambled schematic faces).^7^ If the social-processing-mode hypothesis was true, we would expect to find an attentional bias towards happy face cues only when participants had to classify socially meaningful targets.

Moreover, we made one additional change compared to Experiments 1, 2a, and 2b (and the experiments reported in Wirth & Wentura, 2018a, 2019): In all previous experiments, participants were instructed to search for the stimulus with the double line (or open mouth) in the target display and to ignore the stimulus with a single line (or a closed mouth). In Experiment 3, we counter-balanced this assignment across participants. Thus, one half of the participants had to search for the stimulus containing a single horizontal line (or a closed mouth) while ignoring the stimulus with the double line (or the open mouth). This was done according to general principles of good experimental practice (like balancing key assignments in binary decision tasks). We did not expect any effects with regard to this factor. However, to anticipate it briefly at this point, this manipulation made a difference. While the double-line condition yielded results consistent with the previous experiments, the single-line condition – which was never pretested before – did not work properly.

### Method

#### Participants

Eighty-one university students were compensated for their participation with either €6 or course credit. According to our preregistered outlier criteria, seven participants had to be excluded from all further analyses, because their overall accuracy was more than 3 interquartile ranges below the first quartile of the overall distribution. Notably, all seven outliers were in the single-line target condition.

Of the remaining *N* = 74 participants, 50 were female; age ranged from 18 to 33 years (*M* = 23.3 years, *SD* = 3.9). All participants reported normal or corrected-to-normal vision and provided informed consent prior to testing.

We calculated a power analysis using G*Power, (Faul et al., 2007) based on the lower boundary of the effect sizes that we have found with (upright) happy faces, which ranged from *d*_*z*_ = 0.33 (Experiment 4) 8 to *d*_*z*_ = 0.37 (Experiment 1). In order to detect an effect of size *d*_*z*_ = 0.33 with a power of 1- β = .8 (α = .05), a sample size of 75 participants is required. In order to compensate for potential exclusions of participants, we aimed to test 80 participants in total. The power to detect an effect of *d*_*z*_ = 0.33 with the factual sample size of *N* = 74 was very close to 1 - β = .80 (precisely: .7999, hence the recommendation by G*Power to recruit *N* = 75 participants).

#### Design

We employed a 2 (*cue validity:* valid cue vs. invalid cue) × 2 (*target type:* social target vs. non-social target) × 2 (*target assignment*: double-line target vs. single-line target) design with *cue validity* as a trial-by-trial within-subjects factor, *target type* as a blockwise within-subjects factor, and *target assignment* as between-subjects factor.

#### Materials and procedure

In Experiment 3, the same materials as in Experiment 1 were used. The procedure of Experiment 3 was identical to the procedure of Experiment 1 apart from the following exceptions. In contrast to Experiment 1, the factor *target type* comprised the factor levels social target and non-social target. The social target condition was identical to the select target condition of Experiment 1. For the non-social target condition, scrambled schematic faces were presented. These scrambled faces comprised the same basic features as the schematic faces, but the spatial configuration of those features was altered (i.e., the mouth was located above the nose, one eye and one eyebrow were located above the mouth; see Fig. 2). Thus, the scrambled schematic faces conveyed the impression of a complex, meaningless pattern inside a circle. Participants’ task was to find the target pattern and indicate whether the arrow in this pattern (corresponding to the nose in the social target condition) was pointing up or down. Moreover, participants were told to ignore the arrow in the distractor pattern.

Moreover, participants were randomly assigned to either of two target assignment groups. In the double-line group, the target in the social target condition was defined as the schematic face with an open mouth (as indicated by a double line) and the target in the non-social condition was defined as the pattern that contained a horizontal double line. Conversely, in the single-line group, the target in the social target condition was defined as the schematic face with a closed mouth (as indicated by a single line) and the target in the non-social condition was defined as the pattern that contained a single horizontal line.

### Results

Average classification accuracy was *M* = 95.7% (*SD* = 3.5). Again, the same exclusion criteria were used as in the preceding experiments. This led to the exclusion of 1.9% of all trials with correct responses. After outlier removal, average individual RTs for correct responses ranged from *M* = 588 to *M* = 931 ms (grand mean was *M* = 726 ms, *SD* = 78). Table 4 shows average RTs as a function of the experimental factors.

**Table 4:** Mean response times (RTs) and cueing scores (in ms) of Experiment 3 as a function of target assignment, target type, and cue validity

Target assignment	Target type	Cue validity		
		Valid	Invalid	Cueing score
Double line (*n* = 41)	Social target	712 (96.1)	719 (95.8)	8 [0, 15]
	Non-social target	715 (95.7)	719 (95.8)	4 [-2, 10]
	Overall	714 (95.9)	719 (95.8)	6 [1, 11]
Single line (*n* = 33)	Social target	730 (95.2)	726 (95.4)	-3 [-12, 5]
	Non-social target	753 (95.6)	748 (95.6)	-5 [-14, 4]
	Overall	741 (95.4)	737 (95.5)	-4 [-12, 3]

*Note.* Accuracy rates (in %) are given in parentheses, 95% confidence intervals are given in brackets, cueing score = RT_invalid_ – RT_valid_, deviations between the differences of mean RTs and the cueing scores are due to rounding

We conducted a 2 × 2 × 2 mixed-design ANOVA with the factors *target type* (social target vs. non-social target), *cue validity* (valid cue vs. invalid cue), and *target assignment* (single-line target vs. double-line target) as well as (correct) RTs as the dependent variable. The analysis revealed significant main effects of *target type*, *F*(1, 72) = 4.84, *p* = .031, η_p_^2^ = .063, which reflects faster RTs in the social target condition (*M* = 721 ms, *SD* = 82) than in the non-social target condition (*M* = 732 ms, *SD* = 81). Importantly, there were no significant interaction effects involving *cue validity* and *target type*, all *F*s < 1. Instead, there was a significant *cue validity* × *target assignment* interaction*, F*(1, 72) = 5.21, *p* = .025, η_p_^2^ = .067.

In order to scrutinize this interaction, we calculated cueing scores for both participant groups separately. As can be seen in Table 4, cueing scores in the double-line target group (*M* = 6 ms, *SE* = 2) differed significantly from zero, *t*(40) = 2.26, *p* = .029, *d*_*Z*_ = 0.35, while cueing scores in the single line target group (*M* = -4 ms, *SE* = 4) did not, *t*(32) = 1.14, *p* = .262, *d*_*Z*_ = 0.20.

Although the *target type* × *cue validity* × *target assignment* interaction was not significant, we tested – in anticipation of the discussion – whether the cueing scores for social targets and non-social targets within the double-line group (which are depicted in Fig. 3c) differed significantly. This was not the case, *t*(40) = 0.81, *p* = .422, *d*_*Z*_ = 0.13. As expected, participants’ trait anxiety as assessed with the STAI did not significantly correlate with cueing scores in the single-line target group, *r*(31) = .122, *p* = .498, in the double-line target group, *r*(39) = -.186, *p* = .245, or across both groups, *r*(72) = -.033, *p* = .777.

#### Exploratory analyses

Since the target assignment factor (which was only introduced as a technical factor) unexpectedly moderated the cueing effect, we conducted post hoc analyses to investigate potential reasons for the absence of cueing effects in the single-line target condition (which was not part of the previous experiments). This condition is conspicuous with regard to general performance characteristics. To begin with, the single-line task seemed to be more difficult than the usual double-line task since classification accuracy was significantly lower (in the full sample of *N* = 81) in the former group than in the latter, *t*(79) = 2.71, *p* = .008, *d*_*S*_ = 0.60. Consequently, all of the seven participants that had to be excluded according to our a priori criteria stemmed from the single-line group.

Moreover, in the full sample of *N* = 81 participants, target-distractor congruency effects on accuracy (i.e., the error rate for trials in which the distractor stimulus suggests a different response than the target stimulus minus the error rate for response-congruent trials; see also results section of Experiment 1) were significantly larger in the single-line group than in the double-line group, *t*(79) = 2.36, *p* = .021, *d*_*s*_ = 0.52 (*t*(72) = 1.82, *p* = .072, *d*_*s*_ = 0.43, after exclusion of the accuracy outliers). This suggests that some participants in the single-line group frequently responded – against instructions – to the distractor stimulus with the double line. Again, the overall effect of target-distractor congruency on RTs was significant, *t*(73) = 6.19, *p* < .001, *d*_*z*_ = 0.72.

### Discussion

In Experiment 3, we investigated whether attentional bias towards happy faces is contingent on the activation of a social-processing mode. Participants again performed a dot-probe task where they had to classify either socially meaningful target stimuli (schematic faces) or socially meaningless target stimuli (scrambled schematic faces). If we focus on the double-line target condition (which uses the same target-distractor assignment as previous experiments by Wirth & Wentura, 2018a, 2019, and the present Experiments 1, 2a, and 2b), participants showed a significant attentional bias towards happy face cues. Unlike attentional bias towards angry faces (Wirth & Wentura, 2019), however, this bias was not significantly moderated by the social character of the target stimuli. Thus, it seems that the attentional bias towards happy face cues is not contingent on the activation of a social-processing mode. Nevertheless, it should be noted that the attentional bias towards happy faces was numerically smaller in the non-social target condition. This aspect of the results is discussed in more detail in the General discussion.

Unfortunately, no attentional bias towards happy face cues occurred in the single-line group. However, it seemed to be more difficult for participants in the single-line group to complete the task as response accuracy was significantly lower than in the double-line group. One potential explanation for the increased difficulty is that participants automatically tend to treat the double-line stimulus as the target, potentially because of larger salience of the double line. This explanation was supported by an explorative post hoc analysis based on target-distractor congruency: In the initial sample, the single-line group showed an increased congruency effect on accuracy compared to the double-line group. This result indicates that at least some participants often responded – against instructions – to the double-line stimulus. Consistent with this assumption, removing those participants that were accuracy outliers according to a priori criteria reduced the difference in the congruency effect. This result pattern is consistent with the assumption that the double line might be more salient than the single line: If the double-line stimuli capture spatial attention, this effect will not alter the assumed processes in the invalid cue condition of the single-line target group: Attention is shifted to the happy face and therefore at the location subsequently occupied by the double-line distractor. The time-consuming switch of attention towards the target should be comparable to the invalid condition of the double-line target group. In the valid cue condition, attention is already at the position of the single-line target. However, attentional capture by the double-line distractor will be either executed (with a costly return to the target location) or has to be suppressed. Thus, the assessment of the cueing effect caused by the happy face is severely contaminated.

## Experiment 4

In Experiment 4, we aimed to replicate the attentional bias towards happy face cues that was found in Experiments 1 and 3. Furthermore, Experiment 4 aimed to investigate whether the attentional bias towards happy faces also occurred when another emotional expression of high relevance for the observer was presented during the experiment. One could argue that happy face cues only capture visual attention if they are the most relevant stimulus class presented during the experiment (i.e., if no stimuli of equal or even larger relevance to the observer are presented). For example, basic attention research has shown that attentional capture by color is based on relative information rather than absolute feature values (Becker, Folk, & Remington, 2013). Therefore, in Experiment 4, 50% of the trials contained an angry face (instead of a happy face) and a neutral face in the cue display. We chose angry faces as “competing” emotional stimulus class since anger expressions are of clear relevance to the observer (since they signal immediate threat to the observer). Moreover, we found significant attentional biases towards angry faces under identical conditions in previous experiments (Wirth & Wentura, 2018a, 2019).

### Method

#### Participants

Eighty-four students received course credit as compensation for their participation. The data of one participant were excluded from all further analyses because their average RT was more than three interquartile ranges above the third quartile of the distribution of all participants (Tukey, 1977). Of the remaining *N* = 83 participants, 65 were female; age ranged from 18 to 34 years (*M* = 21.9, *SD* = 3.5).

Again, we aimed to have sufficient power to reveal an attentional bias towards happy (and potentially also angry) face cues. A sample size of *N* = 83 allowed us to detect cueing effects with a size of *d*_*Z*_ = 0.28 with a probability of 1 - β = .80, given an α-value of .05 (one-tailed).

#### Design

We employed a 2 (*cue emotion:* happy vs. angry) × 2 (*cue validity:* valid cue vs. invalid cue) design with both *cue emotion* and *cue validity* as trial-by-trial within-subjects factors.

#### Materials

As happy and neutral face cues, we used the same photographs from the NimStim set of facial expressions (Tottenham et al., 2009) as in Experiment 1. Additionally, we took photographs from the same 16 individuals showing angry expressions. As for happy expressions, we only employed angry faces with non-exposed teeth in Experiment 4. Thus, the intensity of the emotional expression is rather moderate in these faces. Using Adobe Photoshop (Adobe Systems Inc., San Jose, CA, USA), all stimuli were cropped into a standard oval shape concealing hair and external features and were converted to grayscale.

#### Procedure

The procedure of Experiment 4 was identical to the procedure of Experiment 1 apart from the following exceptions. In contrast to Experiment 1, *target type* was not experimentally varied in Experiment 4. Consequently, there was only one experimental block with select targets (i.e., socially meaningful targets accompanied by a distractor). Instead, *cue emotion* was experimentally varied. Thus, on 50% of the trials a happy face and a neutral face were presented on the cue display whereas an angry face and a neutral face were presented on the remaining 50% of the trials. Trials with happy and angry face cues were randomly intermixed (see Fig. 2).

The whole experimental routine again comprised 448 trials. Every 112 trials, a self-paced break was included. At the beginning of the procedure, participants were presented with 32 training trials that were not included in data analysis. At the end of the procedure, participants completed the State-Trait Anxiety Depression Inventory (STADI; Laux, Hock, Bergner-Köther, Hodapp, & Renner, 2013). We decided to administer this updated version of the of the German version of the STAI because it allows to calculate separate scores for anxiety and depression. While there is a strong research tradition linking trait anxiety to attentional bias towards angry faces, one might argue that attentional bias towards happy faces would more likely be (negatively) correlated with trait depression.^9^

### Results

Average classification accuracy was *M* = 96.1% (*SD* = 2.9). For the RT analysis, RTs below 150 ms were excluded, as were RTs more than 1.5 interquartile ranges above the third quartile of the individual participant’s distribution (Tukey, 1977). This led to the exclusion of 1.8% of all trials with correct responses. After outlier exclusion, average individual response times for correct responses ranged from *M* = 607 to *M* = 940 ms (grand mean was *M* = 745 ms, *SD* = 75). Table 5 shows average RTs as a function of the experimental factors.

**Table 5:** Mean response times (RTs) and cueing scores (in ms) of Experiment 4 as a function of target type and cue validity

Cue emotion	Cue validity		
	Valid	Invalid	Cueing score
Happy	741 (96.2)	748 (96.0)	7 [2, 12]
Angry	744 (96.1)	747 (96.2)	3 [-2, 8]
Overall	742 (96.1)	747 (96.1)	5 [2, 8]

*Note.* Accuracy rates (in %) are given in parentheses, 95% confidence intervals are given in brackets, cueing score = RT_invalid_ – RT_valid_, deviations between the differences of mean RTs and the cueing scores are due to rounding

We conducted a 2 × 2 within-subjects ANOVA with the factors *cue emotion* (happy vs. angry) and *cue validity* (valid cue vs. invalid cue), and (correct) RTs as the dependent variable. The analysis revealed a significant main effect of *cue validity*, *F*(1, 82) = 9.19, *p* = .003, η_p_^2^ = .101, which reflects faster RTs for valid trials (*M* = 742 ms, *SD* = 75) than for invalid trials (*M* = 747 ms, *SD* = 77).

The *cue emotion* × *cue validity* interaction did not reach significance, *F*(1, 82) = 1.46, *p* = .230, η_p_^2^ = .018. Nevertheless, for the sake of completeness, we calculated separate cueing scores for happy and angry faces. These cueing scores are depicted in Fig. 3d. Holm-Bonferroni corrected t-tests showed that only cueing scores for happy faces (*M* = 7, *SE* = 2) were significantly different from zero, *t*(82) = 3.02, *p* = .003, *d*_*Z*_ = 0.33, while cueing scores for angry faces (*M* = 3, *SE* = 2) were not, *t*(82) = 1.30, *p* = .196, *d*_*Z*_ = 0.14. The size of the combined cueing effect across both conditions was *d*_*Z*_ = 0.33. As expected, neither the depression score nor the anxiety score nor the global score of the STADI correlated with any of the calculated cueing scores, all |*r*s| < .144, all *p*s > .195.

### Discussion

In Experiment 4, we investigated whether the attentional bias towards happy faces would also occur when angry faces, which also convey a signal of high relevance to the observer, are presented during the experiment. Since a significant attentional bias towards happy faces occurred, the results of Experiment 4 show that the bias is robust even when another highly relevant, frequently attention-capturing emotional expression is presented during the experimental procedure. Furthermore, there was no significant interaction of cue emotion and cue validity, which suggests that the attentional bias towards happy and angry faces is equally large.

Admittedly, the cueing effect for angry faces was not significant when tested separately. This reduced robustness of the bias towards angry faces compared to the bias towards happy faces could reflect that happy faces are more relevant to (non-anxious) observers than angry faces. There is, however, an alternative explanation for this result pattern. Throughout Experiment 4, participants had to focus on the mouth region of the schematic faces to identify the target. The mouth region of a face is more diagnostic for the recognition of happiness than for the recognition of anger. Therefore, a relatively more robust bias towards happy faces might have occurred in Experiment 4.

## General discussion

In the present study, we investigated whether attentional bias towards happy faces occurs in the dot-probe task. To this end, we conducted five experiments that used a short CTOA of 100 ms and perceptually non-confounded happy face cues (without exposed teeth) in order to increase the likelihood of detecting a potential bias. In previous studies (Wirth & Wentura, 2018a, 2019), we showed that (non-anxious) participants show an attentional bias towards angry faces in the dot-probe task only if (1) target stimuli have to compete for attention with simultaneously presented distractor stimuli (i.e., if participants are not in an onset-singleton search mode) and (2) a social-processing mode is activated due to current task demands. Consequently, we additionally investigated in the present experiments whether attentional bias towards happy faces is contingent on similar top-down processes. More specifically, Experiment 1 aimed at investigating whether an attentional bias towards happy faces in the dot-probe task occurs only if targets are accompanied by distractor stimuli (and are thus no onset singletons). Participants performed a dot-probe task with two different target types. In the onset target condition, only a stand-alone schematic target face was presented. In contrast, in the select target condition, the schematic target face was accompanied by a schematic distractor face. Thus, before being able to classify the target face, participants had to select the correct stimulus. The target display was preceded by a cue display that always contained two photographic face cues, one happy and one neutral. We found a significant attentional bias towards happy face cues across both conditions that was not significantly moderated by target type. Thus, the results of Experiment 1 suggest that an attentional bias towards happy faces can occur in the dot-probe task. However, this bias seems to occur regardless of whether participants are searching for targets that are accompanied by distractors or onset-singleton targets.

Since we found a reliable bias towards happy faces in Experiment 1 but several previous studies did not (Baum et al., 2013; Bradley et al., 1997; Cooper & Langton, 2006; Klumpp & Amir, 2009; Mogg & Bradley, 1999; Pourtois et al., 2004; Puls & Rothermund, 2018), one could argue that the happy faces we employed in our study were simply more salient than their neutral counterparts due to low-level perceptual stimulus confounds. This explanation seems unlikely since we only included happy faces with concealed teeth in Experiment 1. Nevertheless, we conducted Experiments 2a and 2b as a control to rule out this possibility. Experiments 2a and 2b were identical to Experiment 1, but inverted face cues were presented throughout the procedure. If the bias towards happy faces found in Experiment 1 was merely caused by low-level stimulus characteristics, we would expect to find a significant bias also towards inverted happy faces. As expected, we found no evidence for an attentional bias towards inverted happy faces.

In Experiment 3, we investigated whether attentional bias towards happy faces in the dot-probe task is contingent on the activation of a social-processing mode. To this end, participants performed a dot-probe task where they had to classify either socially meaningful (schematic faces) or socially meaningless (scrambled schematic faces) target stimuli. Those participants with a target-distractor assignment similar to the previous experiments (participants of the double-line group) again showed an attentional bias towards happy face cues. This bias was, however, not moderated by the social character of the target stimuli.

Unexpectedly, no attentional bias towards happy face cues occurred for participants in the double-line group. As extensively discussed in the Discussion section of Experiment 3, the bias might have been absent in this group because responding to a single-line target unexpectedly seemed to be a more difficult task than responding to a double-line target. Exploratory analyses showed that participants seemed to have larger difficulties to ignore a double-line distractor (if the single-line stimulus had to be categorized) than to ignore a single-line distractor (if the double-line stimulus had to be categorized).

We conducted Experiment 4 for two purposes. First, we aimed to corroborate the occurrence of the bias towards happy faces by a replication in an additional sample of participants. Second, we aimed to investigate whether the bias towards happy faces was moderated by the presentation of faces showing another emotional expression of high relevance (anger) during the experimental procedure. We found an attentional bias towards emotional (angry and happy) faces that was not moderated by emotional expression. Thus, the attentional bias towards happy faces does not seem to be eliminated by the presence of another highly relevant emotional expression.

Taken together, these results show that an attentional bias towards happy faces does occur in the dot-probe task In contrast to attentional bias towards angry faces (Wirth & Wentura, 2018a, 2019), the bias towards happy faces does not seem to be contingent on specific top-down processes – neither on the competition between target and distractor stimuli for attention nor on the activation of a social-processing mode. These findings are compatible with the assumption that any stimuli that are of relevance to the observer (both positive and negative) capture visual attention (e.g., Brosch et al., 2011; Pool et al., 2016) since happy expressions can signal a variety of intentions that are relevant to the observer (e.g., affiliation, safety, and even sexual attraction).

However, previous studies usually did not find attentional biases towards happy faces (e.g., Baum et al., 2013; Bradley et al., 1997; Cooper & Langton, 2006; Klumpp & Amir, 2009; Mogg & Bradley, 1999; Pourtois et al., 2004; Puls & Rothermund, 2018). Thus, even proponents of the relevance-captures-attention hypothesis (Brosch et al., 2008) claimed that happy faces do not produce reliable attentional biases, possibly because happy expressions are simply not sufficiently relevant to observers.

Therefore, one could argue that the cueing effects for happy faces found in the present experiments do not reflect an attentional bias towards happy faces. An alternative explanation would be that these cueing effects were caused by cue-target similarity effects. In most of the experiments reported in this study, we used neutral schematic faces as target stimuli; thus, one might tentatively argue that the change between the cue stimulus and the target stimulus is larger at the position of the happy cue than at the position of the neutral face cue. If so, it is possible that this more noticeable change between cue and target captures visual attention and not the happy expression of the face cues per se. However, this explanation can only be plausibly applied to the select target conditions, but we also found a cueing effect for happy faces in the onset target condition of Experiment 1. A variant of this explanation would be to assume that cue-target similarity effects trigger non-attentional response related processes that accelerate or decelerate participants’ responses. However, the assumption that cue-target similarity effects cause a response bias seems somewhat far-fetched from a theoretical perspective. Moreover, one detail of our results additionally argues against this interpretation: In the non-social target condition of Experiment 3, participants had to classify scrambled schematic faces as targets (which conveyed the impression of a meaningless complex pattern). These scrambled faces were equally similar (or rather dissimilar) to happy and neutral photographic face cues. Nevertheless, participants (at least in the double-line group) showed an attentional bias towards happy face cues in this condition.

Why did we then find an attentional bias towards happy faces? One explanation might be the consistent use of a cue-target onset-asynchrony of 100 ms. Although short CTOAs are recommended by numerous researchers to investigate shifts in covert attention (Cooper & Langton, 2006; Petrova et al., 2013; Stevens et al., 2011; Weierich et al., 2008), many dot-probe studies use longer CTOAs of typically 500 ms (see Chapman, Devue, & Grimshaw, 2017; Cooper & Langton, 2006, for a critical discussion of this standard). Therefore, the attentional bias might simply be too transient to be detectable at 500 ms after cue onset.

Moreover, there might be a less technical reason why many previous studies did not find an attentional bias towards happy faces. In contrast to the present study, many previous dot-probe studies used facial expressions with exposed teeth. We used happy faces with concealed teeth (i.e., closed mouths) in order to avoid any perceptual confounds in our stimuli. However, happy expressions with concealed teeth are not only perceptually less salient, the emotional expression is usually also perceived to be less intense. While roaring laughter (with exposed teeth) usually occurs spontaneously and therefore does not convey specific social signals, these signals are often conveyed by less intense expressions of happiness or pleasure, like subtle smiles or smirks (see also Wirth & Wentura, 2018b, for a study showing that teeth exposure in angry faces can be detrimental to dot-probe effects). While this interpretation seems plausible from the current standpoint, it will have to be corroborated by future research.

inally, we should point out that – although we did not find a significant moderation of the attentional bias by target type (social vs. non-social) in Experiment 3 – the more robust effect was obtained in the social target condition (see Table 4 and Fig. 3). Thus, it is possible that a social-processing mode is indeed a necessary precondition for the occurrence of an attentional bias towards happy faces and that the use of socially meaningful target stimuli is one way to reliably activate such a processing mode. However, the reverse might not be true: Using non-social target stimuli might not be sufficient to guarantee that participants are not in a social-processing mode (e.g., because they are socially processing the cues although they are irrelevant to the task). Again, this assumption has to be corroborated by future research.

Taken together, the present study shows that an attentional bias towards happy faces can occur in the dot-probe task. This bias cannot be explained by perceptual low-level characteristics of the faces used in our experiments. Moreover, the bias towards happy faces seems to be robust as it is not eliminated by the presentation of another highly relevant facial expression (anger) during the experimental procedure. These results are consistent with the assumption that human visual attention is not only biased towards threatening stimuli, but towards potentially relevant stimuli in general.

### Open practices statement

The data and the program code for all experiments are available on the Open Science Framework (OSF). These files can be accessed via the following link: https://osf.io/j5t8u/

Experiments 2a, 2b, and 3 of the present study were preregistered at aspredicted.org. The documentations of these preregistrations can be accessed via the following links:

Experiment 2a: https://aspredicted.org/iw57t.pdf

Experiment 2b: https://aspredicted.org/xn5zp.pdf

Experiment 3: https://aspredicted.org/2bh34.pdf

The stimulus materials of the present study were taken from the NimStim set of facial expressions (Tottenham et al., 2009). Therefore, due to copyright issues, the materials cannot be made publicly available.

## Figures and Tables

**Fig. 4:**
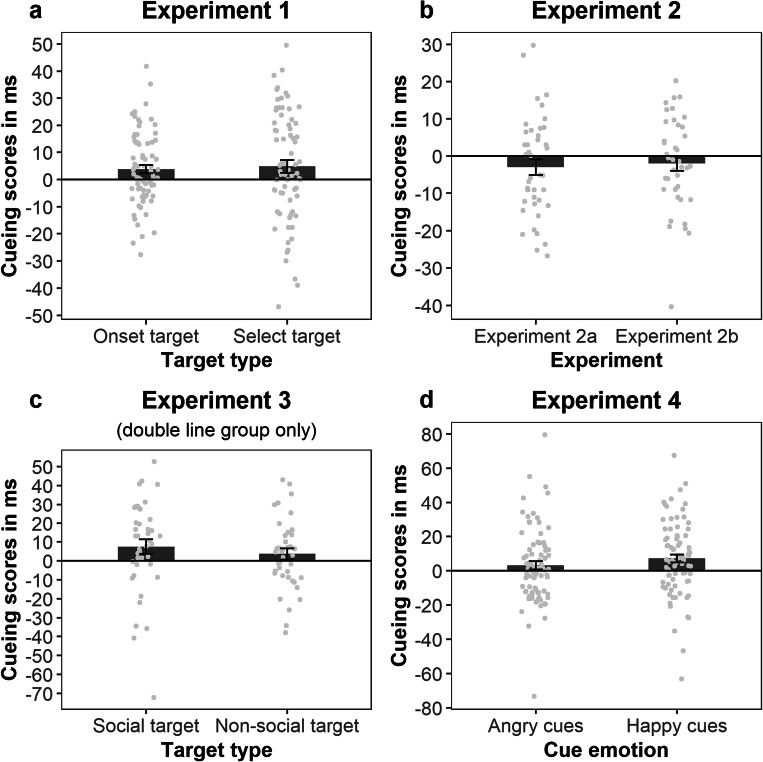
Individual cueing scores (RT_invalid_ – RT_valid_) in all experiments. Bars represent the mean cueing scores of the respective conditions, error bars depict ±1 standard error of the mean (SEM) and points represent cueing scores of individual participants

## Footnotes

1 Throughout the manuscript, we use the terms “valid” and “invalid” in the following sense: A valid cue is a cue that on a given trial appears in the same position as the target. An invalid cue, on the other hand, is a cue that appears in a different position than the target on a given trial. Importantly, referring to a cue as “valid” does not mean that across trials, cue positions are predictive regarding the target positions. That is, cue positions and target positions can be absolutely uncorrelated. Thus, in the case of two potential target locations, 50% of the cues would be valid, while 50% of the cues would be invalid.

2 One participant accidentally did not complete the STAI at the end of the procedure. Therefore, these analyses included only 73 participants (hence the reduced number of *df*s).

3 We pre-registered our planned procedure on aspredicted.org. The pre-registration can be accessed via the following link: https://aspredicted.org/iw57t.pdf

4 While we identified and excluded outliers in Experiment 1 based on the distributions of the accuracy and RT distributions in the final sample (see Tukey, 1977), this procedure is not a viable option when sequential Bayes factors are used (because there simply is no final sample). Therefore, we used the absolute cut-off values of Experiment 1 for exclusion. These absolute cut-off values were accuracies lower than 88.9% or 84.4% in the onset target condition or the select target condition, respectively, or average RTs slower than 879 ms or 1,128 ms in the onset target condition or the select target condition, respectively. This exclusion rationale was also documented in the pre-registration.

5 Again, we pre-registered our planned procedure (including the exclusion criteria, see below) on aspredicted.org. The pre-registration can be accessed via the following link: https://aspredicted.org/xn5zp.pdf

6 Although the cueing score was again slightly negative (*M* = -2 ms, *SD* = 12), the cueing score was not significant according to NHST, *t*(39) = 1.01, *p* = .319, *d*_z_ = 0.16.

7 The procedure and analyses of Experiment 3 were pre-registered on aspredicted.org. The pre-registration can be accessed via the following link: https://aspredicted.org/2bh34.pdf

8 Experiment 4 of the present article was conducted before Experiment 3.

9 For the sake of transparency, it should be mentioned that in the context of a student project, the same sample also completed the German version of the Positive and Negative Affect Schedule (PANAS; Krohne, Egloff, Kohlmann, & Tausch, 1996) and the German version of the Toronto Alexithymia Scale (TAS – 26; Kupfer, Brosig, & Brähler, 2001) at the end of the procedure. However, these measures did not correlate with attentional bias towards happy or angry faces all |*r*s| < .100, all *p*s > .370.
